# Risk Factors for Intracerebral Hemorrhage in Patients With Atrial Fibrillation on Non–Vitamin K Antagonist Oral Anticoagulants for Stroke Prevention

**DOI:** 10.1161/STROKEAHA.120.031827

**Published:** 2021-03-04

**Authors:** Maurizio Paciaroni, Giancarlo Agnelli, Michela Giustozzi, Valeria Caso, Elisabetta Toso, Filippo Angelini, Isabella Canavero, Giuseppe Micieli, Kateryna Antonenko, Alessandro Rocco, Marina Diomedi, Aristeidis H. Katsanos, Ashkan Shoamanesh, Sotirios Giannopoulos, Walter Ageno, Samuela Pegoraro, Jukka Putaala, Daniel Strbian, Hanne Sallinen, Brian Mac Grory, Karen L. Furie, Christoph Stretz, Michael E. Reznik, Andrea Alberti, Michele Venti, Maria Giulia Mosconi, Maria Cristina Vedovati, Laura Franco, Giorgia Zepponi, Michele Romoli, Andrea Zini, Laura Brancaleoni, Letizia Riva, Giorgio Silvestrelli, Alfonso Ciccone, Maria Luisa Zedde, Elisa Giorli, Maria Kosmidou, Evangelos Ntais, Lina Palaiodimou, Panagiotis Halvatsiotis, Tiziana Tassinari, Valentina Saia, Raffaele Ornello, Simona Sacco, Fabio Bandini, Michelangelo Mancuso, Giovanni Orlandi, Elena Ferrari, Alessandro Pezzini, Loris Poli, Manuel Cappellari, Stefano Forlivesi, Alberto Rigatelli, Shadi Yaghi, Erica Scher, Jennifer A. Frontera, Luca Masotti, Elisa Grifoni, Pietro Caliandro, Aurelia Zauli, Giuseppe Reale, Simona Marcheselli, Antonio Gasparro, Valeria Terruso, Valentina Arnao, Paolo Aridon, Azmil H. Abdul-Rahim, Jesse Dawson, Carlo Emanuele Saggese, Francesco Palmerini, Boris Doronin, Vera Volodina, Danilo Toni, Angela Risitano, Erika Schirinzi, Massimo Del Sette, Piergiorgio Lochner, Serena Monaco, Marina Mannino, Rossana Tassi, Francesca Guideri, Maurizio Acampa, Giuseppe Martini, Enrico Maria Lotti, Marina Padroni, Leonardo Pantoni, Silvia Rosa, Pierluigi Bertora, George Ntaios, Dimitrios Sagris, Antonio Baldi, Cataldo D’Amore, Nicola Mumoli, Cesare Porta, Licia Denti, Alberto Chiti, Francesco Corea, Monica Acciarresi, Yuriy Flomin, Nemanja Popovic, Georgios Tsivgoulis

**Affiliations:** Stroke Unit and Division of Cardiovascular Medicine, University of Perugia, Italy (M. Paciaroni, G.A., M.G., V.C., A.A., M.V., M.G.M., M.C.V., L.F., G.Z.).; Division of Cardiology, Città della Salute e della Scienza Hospital, University of Torino, Italy (E.T., F.A.).; Emergency Neurology, IRCCS Casimiro Mondino Foundation, Pavia, Italy (I.C., G. Micieli).; Department of Neurology, Bogomolets National Medical University, Kyiv, Ukraine (K.A.).; Stroke Unit, Department of Systems Medicine, University of Tor Vergata, Rome, Italy (A. Rocco, M.D.).; Department of Medicine (Neurology), McMaster University/Population Health Research Institute, Hamilton, Canada (A.H.K., A.S.).; Department of Neurology, University of Ioannina School of Medicine, Greece (S.G., E.N.).; Department of Medicine, University of Insubria, Ospedale di Circolo, Varese, Italy (W.A., S.P.).; Department of Neurology, Helsinki University Hospital and Neurosciences University of Helsinki, Finland (J.P., D. Strbian, H.S.).; Department of Neurology, Duke University School of Medicine, Durham, NC (B.M.G.).; Department of Neurology, Warren Alpert Medical School of Brown University, Providence, RI (K.L.F., C.S., M.E.R.).; Neurology Unit, Rimini “Infermi” Hospital–AUSL Romagna, Rimini, Italy (M. Romoli).; Department of Neurology and Stroke Center, IRCCS Istituto di Scienze Neurologiche di Bologna (A. Zini, L.B.), Maggiore Hospital, Bologna, Italy.; Division of Cardiology (L.R.), Maggiore Hospital, Bologna, Italy.; S.C. di Neurologia e S.S. di Stroke Unit, ASST di Mantova, Italy (G.S., A. Ciccone).; Neurology Unit, Stroke Unit, Local Health Unit–IRCCS of Reggio Emilia, Italy (M.L.Z.).; Department of Neurology, Stroke Unit, Sant’Andrea Hospital, La Spezia, Italy (E. Giorli).; First Department of Internal Medicine, University of Ioannina School of Medicine, Greece (M.K.).; Second Department of Neurology, “Attikon” Hospital, National and Kapodistrian University of Athens, School of Medicine, Greece (L. Palaiodimou, G.T.).; Second Department of Internal Medicine “Attikon” University Hospital Medical School, National and Kapodistrian University of Athens, Greece (P.H.).; Department of Neurology and Stroke Unit, Santa Corona Hospital, Pietra Ligure (Savona), Italy (T.T., V.S.).; Neuroscience Section, Department of Applied Clinical Sciences and Biotechnology, University of L’Aquila, Italy (R.O., S.S.).; Department of Neurology, Ospedale San Paolo, Savona, Italy (F.B.).; Department of Clinical and Experimental Medicine, Neurological Institute, University of Pisa, Italy (M. Mancuso, G.O., E.F.).; Department of Clinical and Experimental Sciences, Neurology Unit, University of Brescia, Italy (A.P., L. Poli).; SSO Stroke Unit, UO Neurologia, DAI di Neuroscienze, AOUI Verona, Italy (M.C., S.F.).; Pronto Soccorso, Ospedale Borgo Trento, DAI Emergenza e Accettazione, AOUI Verona, Italy (A. Rigatelli).; Department of Neurology, NYU Langone Health, New York, NY (S.Y., E. Scher, J.A.F.).; Internal Medicine, San Giuseppe Hospital, Empoli, Italy (L.M., E. Grifoni).; Neurology Unit, Fondazione Policlinico Universitario A. Gemelli IRCCS, Rome, Italy (P.C.).; Department of Geriatrics, Neurosciences and Orthopedics, Università Cattolica del Sacro Cuore, Rome, Italy (A. Zauli, G.R.).; Humanitas Clinical and Research Center–IRCCS, Rozzano, Milano, Italy (S. Marcheselli).; Neurologia, Ospedali Riuniti, Palermo, Italy (A.G., V.T.).; Department of Biomedicine, Neuroscience and Advanced Diagnostics, University of Palermo, Italy (V.A., P.A.).; Medical School and Institute of Cardiovascular and Medical Sciences, University of Glasgow, United Kingdom (A.H.A.-R., J.D.).; Unità di Terapia Neurovascolare, Ospedale “Fabrizio Spaziani,” Frosinone, Italy (C.E.S.).; Istituto Ospedaliero Fondazione Poliambulanza, Brescia, Italy (F.P.).; Municipal Budgetary Healthcare Institution of Novosibirsk, City Clinical Hospital No. 1, Novosibirsk State Medical University, Russia (B.D., V.V.).; Department of Human Neurosciences, Sapienza University of Rome, Italy (D.T., A. Risitano).; Struttura Complessa di Neurologia, Ente Ospedaliero Ospedali Galliera, Genoa, Italy (E. Schirinzi, M.D.S.).; Department of Neurology, Saarland University, Medical Center, Homburg, Germany (P.L.).; Stroke Unit, Ospedale Civico, Palermo, Italy (S. Monaco, M. Mannino).; Stroke Unit, AOU Senese, Siena, Italy (R.T., F.G., M. Acampa, G. Martini).; U.O. Neurologia Presidio Ospedaliero di Ravenna Azienda USL della Romagna, Italy (E.M.L., M. Padroni).; ‘L. Sacco’ Department of Biomedical and Clinical Sciences, University of Milan, Italy (L. Pantoni, P.B.).; Neurology Unit, ASST Fatebenefratelli–Sacco, Milan, Italy (S.R.).; Department of Medicine, University of Thessaly, Larissa, Greece (G.N., D. Sagris).; Stroke Unit, Ospedale di Portogruaro, Venice, Italy (A.B., C.D.).; Department of Internal Medicine, Magenta Hospital, Italy (N.M., C.P.).; Stroke Unit, Dipartimento Geriatrico Riabilitativo, University of Parma, Italy (L.D.).; Neurologia, Ospedale Apuano, Massa Carrara, Italy (A. Chiti).; UO Gravi Cerebrolesioni, San Giovanni Battista Hospital, Foligno, Italy (F.C., M. Acciarresi).; Stroke and Neurorehabilitation Unit, MC Universal Clinic ‘Oberig’ Kyiv, Ukraine (Y.F.).; Clinic of Neurology, Clinical Center of Vòsvodina, University of Novi Sad, Serbia (N.P.).

**Keywords:** atrial fibrillation, cerebral hemorrhage, logistic models, risk factors, white matter

## Abstract

**Background and Purpose:**

Clinical trials on stroke prevention in patients with atrial fibrillation have consistently shown clinical benefit from either warfarin or non–vitamin K antagonist oral anticoagulants (NOACs). NOAC-treated patients have consistently reported to be at lower risk for intracerebral hemorrhage (ICH) than warfarin-treated patients. The aims of this prospective, multicenter, multinational, unmatched, case-control study were (1) to investigate for risk factors that could predict ICH occurring in patients with atrial fibrillation during NOAC treatment and (2) to evaluate the role of CHA_2_DS_2_-VASc and HAS-BLED scores in the same setting.

**Methods:**

Cases were consecutive patients with atrial fibrillation who had ICH during NOAC treatment. Controls were consecutive patients with atrial fibrillation who did not have ICH during NOAC treatment. As within the CHA_2_DS_2_-VASc and HAS-BLED scores there are some risk factors in common, several multivariable logistic regression models were performed to identify independent prespecified predictors for ICH events.

**Results:**

Four hundred nineteen cases (mean age, 78.8±8.1 years) and 1526 controls (mean age, 76.0±10.3 years) were included in the study. From the different models performed, independent predictors of ICH were increasing age, concomitant use of antiplatelet agents, active malignancy, high risk of fall, hyperlipidemia, low clearance of creatinine, peripheral artery disease, and white matter changes. Low doses of NOACs (given according to label or not) and congestive heart failure were inversely associated with the risk of ICH. HAS-BLED and CHA_2_DS_2_-VASc scores performed poorly in predicting ICH with areas under the curves of 0.496 (95% CI, 0.468–0.525) and 0.530 (95% CI, 0.500–0.560), respectively.

**Conclusions:**

Several risk factors were associated to ICH in patients treated with NOACs for stroke prevention but not HAS-BLED and CHA_2_DS_2_-VASc scores.

## Abstract 2

Supplemental Digital Content is available in the text.

Clinical trials on stroke prevention in patients with atrial fibrillation (AF) have consistently shown benefits from either warfarin or non–vitamin K antagonist oral anticoagulants (NOACs). However, these patients are known to experience anticoagulation-related intracerebral hemorrhage (ICH).

The aims of this prospective, multicenter, international study were (1) to investigate for risk factors that could predict ICH occurring in AF patients during NOAC treatment in a large multinational cohort of patients across Europe and North America and (2) to evaluate the role of CHA_2_DS_2_-VASc and HAS-BLED scores in the same setting.

## Methods

The data that support the findings of this study are available from the corresponding author on reasonable request.

This was a multicenter unmatched case-control study performed between June 2018 and February 2020. Consecutive patients with AF who experienced an acute nontraumatic ICH while on treatment with NOACs (dabigatran, apixaban, rivaroxaban, or edoxaban) for stroke prevention were included in the study, as well as those patients who had died due to the index event. ICH was classified as deep, when located in the basal ganglia, thalamus, pons, or the cerebellum, and lobar, when located in the brain cortex. Patients with subdural hematoma, epidural hematoma, isolated intraventricular hemorrhage, or subarachnoid hemorrhage were not included in the study.

Patients with ICH, identified as cases, were enrolled from 44 Stroke Units across Europe, United States, and Canada. Controls were patients with AF who had been taking NOACs for stroke prevention for >1 month and did not experience ICH events while on anticoagulant therapy. Controls were consecutive in- and outpatients attending 4 European anticoagulant therapy services (Torino [268 patients], Perugia [1023 patients], Varese [15 patients], and Kyiv [54 patients]) and 10 Stroke Unit follow-up services (Larissa, Ioannina, Athens, La Spezia, Pisa, Pavia, Frosinone, Foligno, Brescia, and Rome Tor Vergata [166 patients]).

To verify compliance, the patients and family members were asked how the prescribed anticoagulant was taken.

The duration of therapy for controls was calculated from the first visit, when the anticoagulant therapy was initiated, and risk factors were collected up to the last visit performed over the study time period.

The study was approved by the pertinent institutional review boards, if required. Informed consent was obtained whenever necessary.

### Risk Factors

For cases at the time of ICH and controls at baseline updated during the follow-up visits, data on known stroke risk factors were collected (Data Supplement).

### Statistical Analysis

The aim of the unmatched analyses was to identify predictors of ICH events. Univariate tests (χ^2^ test or Fisher exact test with Yates correction when appropriate) were used to compare patients with ICH events (cases) with controls, regarding risk factors for cerebrovascular disease.

As within the CHA_2_DS_2_-VASc and HAS-BLED scores there are some risk factors in common, several multivariable logistic regression models were performed to identify independent predictors for ICH events (Data Supplement).

Due to the low number of centers that included control patients with possible selection bias, sensitivity analyses were performed to test the robustness of the results obtained with the multivariable models, restricting the cohort to each single center enrolling >200 controls, independently from cases provided. The first sensitivity analysis compared cases with controls included from the Perugia center; the second sensitivity analysis compared cases with controls included from the Torino center.

Furthermore, receiver operating characteristic curves along with the Mann-Whitney *U* test were used to illustrate the performance of HAS-BLED and CHA_2_DS_2_-VASc scores in predicting ICH.

Data were analyzed with the SPSS/PC Win package 25.0.

### Sample Size Calculation

For this unmatched case-control study, it was assumed that at least 5% of controls would have had the risk factor with the lower incidence. Due to the low prevalence of exposure to ICH among patients with AF during NOAC therapy in real world (0.5% per year), the increase in statistical power was obtained by using a ratio of controls/cases of 3:1.^1,2^ To detect a minimum odds ratio of 2.0 with a power of 90% and an alpha risk of 5%, it was calculated that at least a total of 1776 patients (444 cases and 1332 controls) would have been needed, whereas with a power of 80%, it was calculated that at least a total of 1288 patients (322 cases and 966 controls) would have been needed.

## Results

During the study period, 419 consecutive patients on NOACs were admitted for ICH (cases). The cases were compared with a control group of 1526 subjects (Table). Cases resulted having ICH after an average of 20 months from the initiation of therapy. The characteristics of the patients with deep or lobar ICH are described in Table I in the Data Supplement.

**Table.**
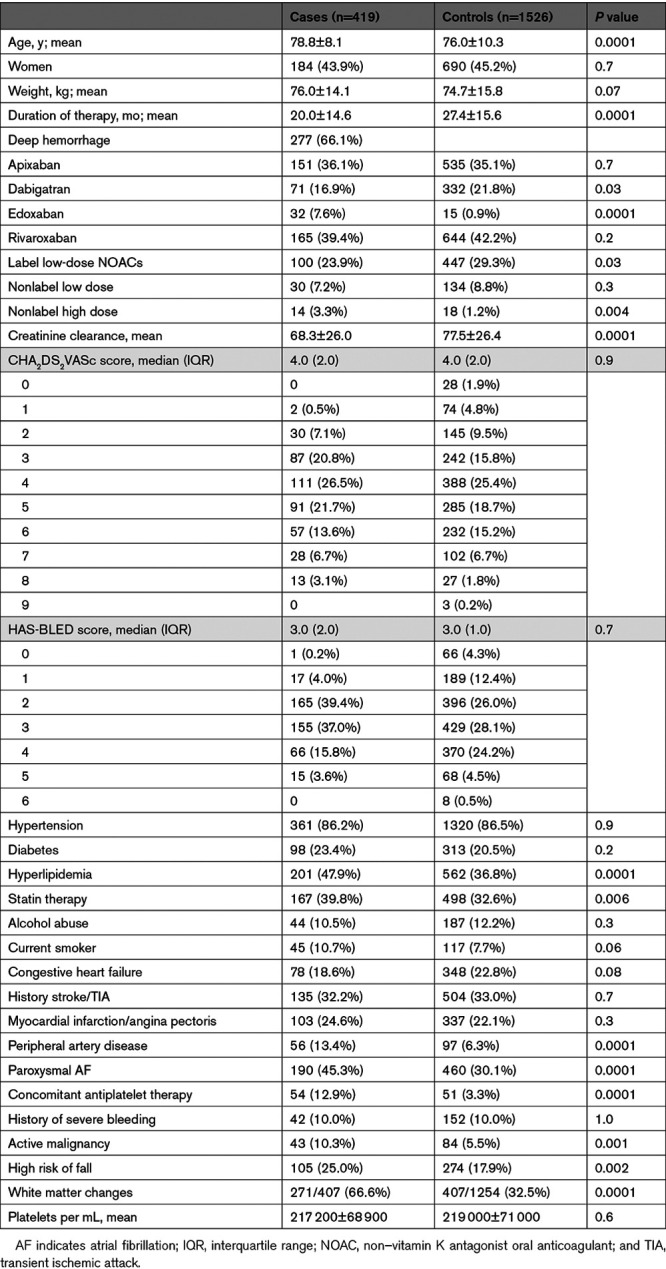
Characteristics of the Cases and Controls

The results of the multivariable analyses and the odds ratio for each variable included using the different models are reported in Tables II through IV in the Data Supplement. Patients treated with low doses (given according to label or not) had a lower risk of ICH, as did patients with congestive heart failure. Age, concomitant use of antiplatelet agents, white matter changes, hyperlipidemia, peripheral artery disease, history of active malignancy, high risk of fall, and low clearance of creatinine were associated with ICH. HAS-BLED (odds ratio, 1.00 [95% CI, 0.90–1.11]) and CHA_2_DS_2_-VASc (odds ratio, 1.02 [95% CI, 0.93–1.11]) scores were not associated with ICH.

The sensitivity analyses confirmed that increasing age, hyperlipidemia, concomitant use of antiplatelets, high risk of fall, white matter changes, and low clearance of creatinine were independently associated with ICH (Tables V through VII in the Data Supplement).

Areas under the curves of the two scores cross over 95% CI: C statistics for HAS-BLED and CHA_2_DS_2_-VASc scores were 0.496 (95% CI, 0.468–0.525) and 0.530 (95% CI, 0.500–0.560), respectively. Also, utilizing the Mann-Whitney *U* test, the two scores were not associated with ICH (*P*=0.8 for HAS-BLED and *P*=0.06 for CHA_2_DS_2_-VASc).

## Discussion

This unmatched case-control study showed that the HAS-BLED and CHA_2_DS_2_-VASc scores performed poorly in predicting ICH, which suggests against the use of these risk scores when assessing the indication for anticoagulation with NOACs.

Our study had the following limitations^1^: it was observational, and neither individual NOACs nor their doses were randomized^2^; other pharmacological treatments besides NOACs except antiplatelets were not investigated. In fact, this limitation might be considered a serious shortcoming, in that it might hinder the interpretation of these data. Interactions between NOACs and other drugs are reported to be much lower than that of warfarin. Specifically, all currently available NOACs are substrates of the P-glycoprotein transporter; one-third of rivaroxaban is metabolized by the liver via CYP3A4/CYP3A5- and CYP2J2-dependent pathways; and apixaban, which has predominant nonrenal clearance, is eliminated via the CYP3A4-, CYP1A2-, and CYP2J2-dependent pathways. Therefore, it is plausible that drug interactions may have interfered with the anticoagulant effect^3^; we excluded patients who could not guarantee adherence to the prescribed treatment regimen. As this information was provided by the patients themselves or the caregiver, a laboratory assessment of the anticoagulant status during the event might have been informative.^3,4^ Cases were collected from a number of Stroke Units in Europe, United States, and Canada. Unfortunately, not all participating Stroke Units had an associated anticoagulant unit where the controls could have been collected. For this reason, we collected control data from 14 centers, all except one, associated to a Stroke Unit and tested the results in a sensitivity analysis including data from large centers only. Regarding this, although potentially limited by the sample size, sensitivity analyses confirmed factors associated with ICH that emerged from multivariate analyses, consequently supporting the robustness of the results. Although we attempted to control by size the included patients, there may still be important differences in regard to follow-up frequency and collection of the data that could at least partially explain the findings.

The strengths of our study include its large sample size and its prospective design. Additionally, our analyses reflect real-life experiences and thus may provide valuable information that could significantly reduce the incidence of ICH in patients with AF and stroke during NOAC therapy.

## Conclusions

In patients with AF treated with NOACs, age, concomitant use of antiplatelet agents, the presence of an active malignancy, high risk of fall, hyperlipidemia, low clearance of creatinine, peripheral artery disease, and white matter changes on neuroimaging were associated with increased risk of ICH. Low doses of NOACs (given according to label or not) and congestive heart failure were inversely associated with the risk of ICH. The HAS-BLED and CHA_2_DS_2_-VASc scores performed poorly in predicting ICH.

## Sources of Funding

None.

## Disclosures

Dr Paciaroni received honoraria as a member of the Speaker Bureau of Aspen, Sanofi-Aventis, Boehringer Ingelheim, Bayer, Bristol Myers Squibb (BMS), Daiichi Sankyo, and Pfizer. Dr Caso received honoraria as a member of the Speaker Bureau of Boehringer Ingelheim, Bayer, and Daiichi Sankyo (all fees were paid to Associazione Ricerca Stroke, Umbria). She received honoraria as a consultant or advisory board member of Boehringer Ingelheim, Bayer, Daiichi Sankyo, and Pfizer. Dr Ntaios received research funding from Pfizer; honoraria from Pfizer, Boehringer Ingelheim, and Bayer; and consultant honoraria from Pfizer, Boehringer Ingelheim, and Bayer. Dr Tsivgoulis has received funding for travel or speaker’s honoraria from Bayer, Pfizer, and Boehringer Ingelheim. He has served on scientific advisory boards for Bayer, Boehringer Ingelheim, and Daiichi Sankyo. Dr Putaala has received personal fees from Boehringer Ingelheim, Bayer, and Portola. He has also received grants and personal fees from BMS/Pfizer and Abbott/St. Jude Medical. Dr Del Sette has received honoraria for speaking from Bayer and Boehringer Ingelheim. Dr Riva has received speaker fees and consulting honoraria for advisory board from Boehringer Ingelheim, Bayer, and Daiichi Sankyo. Dr Stretz has received personal fees from the Neurocritical Care Society. Dr Ornello has received nonfinancial support from Novartis, Allergan, and Teva. Dr Sallinen has received funding from Helsinki University Hospital Research Funds, Maire Taponen Foundation, Biomedicum Helsinki Foundation, and the Finnish Medical Foundation. Dr Ageno has received grants and personal fees from Bayer and personal fees from Boehringer Ingelheim, BMS/Pfizer, Portola, Jansen, Aspen, Sanofi, and Daiichi Sankyo. Dr Sacco has received personal fees and nonfinancial support from Abbott and Allergan, as well as nonfinancial support from Eli Lilly, Novartis, Teva, Bayer, BMS, Daiichi Sankyo, Medtronic, Pfizer, and Starmed. Dr Corea reports having received expenses for meetings from Novartis. Dr Giannopoulos has received funding for travel from Bayer and speaker’s honoraria from Pfizer. Dr Cappellari has received consulting fees from Boehringer Ingelheim, Pfizer-BMS, and Daiichi Sankyo. Dr Dawson reports honoraria as a member of the speaker bureau of Boehringer Ingelheim, Bayer, BMS, Daiichi Sankyo, Medtronic, and Pfizer. He has also received research funding from Pfizer. Dr Zini has received speaker fees and consulting fees from Boehringer Ingelheim, Medtronic, Cerenovus and the advisory boards of Daiichi Sankyo, Boehringer Ingelheim, and Stryker. Dr Toni has received personal fees from Abbott, Bayer, Boehringer Ingelheim, Daiichi Sankyo, Medtronic, and Pfizer. Dr Shoamanesh has received research funding from Daiichi Sankyo, Bayer AG, BMS/Pfizer, Portola Pharmaceuticals, and Octapharma. He has received honoraria from Daiichi Sankyo (consulting), Bayer AG (speaking and consulting), BMS/Pfizer (speaking and consulting), Servier Canada (speaking and consulting), and Boehringer Ingelheim (consulting). Dr Strbian received honoraria from Portola and BMS for participation in scientific advisory board and unrestricted educational grant from Boehringer Ingelheim. Dr Flomin has received personal fees from Boehringer Ingelheim, Bayer, and Takeda; grants, personal fees, and nonfinancial support from Pfizer; and personal fees and nonfinancial support from Sanofi Genzyme. The other authors report no conflicts.

## Supplemental Materials

Methods (continuation)

Statistical Analysis (continuation)

Tables I–VII

## Supplementary Material


