# Supplementary material for: Risk Factors for Intracerebral Hemorrhage in Patients With Atrial Fibrillation on Non–Vitamin K Antagonist Oral Anticoagulants for Stroke Prevention
Source: Stroke. 2021 Mar 4;52(4):1450–4. doi: 10.1161/STROKEAHA.120.031827 (PMC10561687; doi:10.1161/STROKEAHA.120.031827)
Supplement: Supplementary file 1 [file str-52-1450-s001.pdf]

Supplemental material

Methods (continuation)
Risk Factors
Data on known stroke risk factors were collected as following: age, sex, history of hypertension (blood pressure>140/90 mm Hg at least twice before acute stroke or already under treatment with antihypertensive drugs), history of diabetes mellitus (fasting glucose level >126 mg/dL pre-prandial on 2 examinations, glucose level >200 mg/dL postprandial, or glycohemoglobin >6.5%, or under antidiabetic treatment), current cigarette smoking, hyperlipidemia (total cholesterol >200 mg/dL or triglyceride >140 mg/dL or already under lipid-lowering therapy), history of symptomatic ischemic heart disease (myocardial infarction, history of angina or previous diagnosis of multiple lesions on thallium heart isotope scan, or evidence of coronary disease on coronary angiography), history of symptomatic peripheral artery disease (intermittent claudication of presumed atherosclerotic origin, or ankle/arm systolic blood pressure ratio <0.85 in either leg at rest, or history of intermittent claudication with previous leg amputation, reconstructive surgery, or angioplasty), alcohol abuse (>300 g per week), obesity (body mass index >30 kg/m2), or previous stroke/transient ischemic attack. Likewise, baseline variables were obtained for all patients including creatinine clearance (calculated by Cockcroft-Gault equation), type, and duration of NOAC treatment. The doses of NOACs were recorded, and the reasons for prescribing low doses were also collected. Low doses of NOACs were considered off label in the absence of the recommended clinical and laboratory criteria for dose reduction. Low dose of dabigatran was considered as labeled for elderly patients (age ≥80 years), patients with moderate renal impairment (creatinine clearance, 30–49 mL/min), and those with concomitant use of interacting drugs (as verapamil). Low dose of rivaroxaban was considered as labeled for patients with moderate or severe renal impairment (creatinine clearance, 15–49 mL/min). Low-dose apixaban was considered as labeled for patients with at least 2 of the following: age ≥80 years, weight ≤60 kg, or serum creatinine ≥1.5 mg/dL or with severe renal impairment (creatinine clearance 15-29 mL/min). Low-dose edoxaban was considered as labeled in patients with moderate or severe renal impairment (creatinine clearance, 15–49 mL/min), in those with concomitant use of interacting drugs and in those with a weight ≤60 kg. High dose was considered non-label for patients with these same characteristics, but not taking a low dose. Nonvalvular AF was classified as paroxysmal when associated with episodes terminating spontaneously within 7 days.
For cases, both the CHA_2DS_2-VASc score (2 points for history of stroke or age >75 years and 1 point each for congestive heart failure, hypertension, diabetes mellitus, vascular disease, age between 65 and 74 years, and female sex) and the HAS-BLED score [1 point for Hypertension (uncontrolled, >160 mmHg systolic), abnormal renal function (dialysis, transplant, creatinine >2.26 mg/dL or >200 µmol/L), abnormal liver function (cirrhosis or bilirubin >2x normal or AST/ALT/AP >3x normal), prior history of stroke, bleeding (prior major bleeding or predisposition to bleeding), labile INR (unstable/high INR, Time in Therapeutic Range < 60%), age > 65 years, prior alcohol (≥ 8 drinks/week), or medication predisposing to bleeding (antiplatelet agents, NSAIDs)] (8) were calculated before the ICH event. For controls, both the CHA_2DS_2-VASc score and HAS-BLED score were calculated at the time of anticoagulant therapy initiation.
White matter changes (defined on the first computed tomographic [or magnetic resonance] examination as ill-defined and moderately hypodense [or hyperintensity on T2 weighted on magnetic resonance] areas >5 mm according to published criteria) were investigated. White matter changes were dichotomized into absent versus present.
The following variables were also collected: platelet count, previous major bleeding (defined as being intracranial bleedings, hospitalization, hemoglobin decrease > 2 g/dL, and/or transfusion), simultaneous antiplatelet use, presence of malignancy, any prescribed use of statins and high risk of fall (at least one of: a) underlying medical condition such as neurological cardiac or other disabling conditions; b) side effects of medications, physical inactivity and loss of balance, particularly among older people; c) poor mobility, cognition and vision, particularly among those living in an

institution, such as a nursing more or chronic care facility; d) unsafe environments, particularly for those with poor balance and limited vision).

Statistical Analysis

The prespecified variables included in the first model were HAS-BLED score (as continuous variable excluding the risk factors within the score), different doses of NOACs (standard dose as reference), sex, hyperlipidemia, diabetes mellitus, current smoker, history of myocardial infarction, peripheral artery disease, platelet count, therapy with statins, history of active malignancy and high risk of fall.
The prespecified variables included in the second model were CHA_2DS_2-VASc score (as continuous variable excluding the risk factors within the score), different doses of NOACs (standard dose as reference), alcohol abuse, hyperlipidemia, current smoker, creatinine clearance, therapy with statins, platelet count, concomitant antiplatelet therapy, history of major bleeding, history of active malignancy and high risk of fall.
The prespecified variables included in the third model were: different doses of NOACs (standard dose as reference), age, sex, hypertension, diabetes mellitus, hyperlipidemia, history of myocardial infarction, peripheral artery disease, congestive heart failure, history of stroke/TIA, alcohol abuse, creatinine clearance, current smoker, history of major bleeding, therapy with statins, platelet count, concomitant antiplatelet therapy, history of active malignancy and high risk of fall, excluding HAS-BLED and CHA_2DS_2-VASc scores.
A fourth model was performed including the same variables as the third model with the addition of white matter changes, as 1,661 out of 1,945 subjects had undergone a neuroimaging examination.

Table I. Characteristics of the patients with deep or lobar ICH

	Deep (n=277)	Lobar (n=142)	p
Age (years), mean	78.7±8.1	79.2±8.1	0.2
Males	162 (58.5%)	73 (51.4%)	0.1
Apixaban	101 (36.5%)	50 (35.2%)	0.8
Dabigatran	36 (13.0%)	34 (23.9%)	0.004
Edoxaban	19 (6.8%)	13 (9.1%)	0.4
Rivaroxaban	121 (43.7%)	45 (31.7%)	0.01
Hypertension	245 (88.4%)	114 (80.3%)	0.02
Diabetes Mellitus	62 (22.4%)	35 (24.6%)	0.6
Hyperlipidemia	155 (56.0%)	75 (52.8%)	0.5
Alcohol abuse	31 (11.2%)	13 (9.2%)	0.4
Current smoker	28 (10.1%)	17 (12.0%)	0.4
Congestive heart failure	55 (19.8%)	23 (16.2%)	0.3
History stroke/TIA	81 (29.2%)	53 (37.3%)	0.09
Myocardial infarction/angina pectoris	71 (25.6%)	32 (22.5%)	0.4
Peripheral artery disease	37 (13.3%)	19 (13.4%)	1.0
Concomitant antiplatelet therapy	37 (13.3%)	17 (12.0%)	0.8
History of severe bleeding	27 (9.7%)	15 (10.6%)	0.6
Active malignancy	26 (9.4%)	17 (12.0%)	0.2
High risk of fall	74 (26.7%)	31 (21.8%)	0.2
White matter changes	190 (68.6%)	79 (55.6%)	0.008

Table II. Multivariable analysis (model including HAS-BLED score): predictive factors for intracerebral hemorrhage

	OR (95% CI)	P
Standard dose	Reference	
Non-label high dose NOACs	1.76 (0.80-3.88)	0.1
Label low dose	0.74 (0.56-0.91)	0.03
Non-label low dose	0.61 (0.38-0.98)	0.04
HAS-BLED score	1.00 (0.90-1.11)	0.9
Females	0.95 (0.75-1.22)	0.6
Hyperlipidemia	2.23 (1.71-2.90)	0.0001
Diabetes mellitus	1.09 (0.82-1.43)	0.5
Current smoker	1.18 (0.80-1.76)	0.3
History of myocardial infarction	1.07 (0.81-1.42)	0.6
Peripheral artery disease	2.18 (1.50-3.17)	0.0001
Platelet count	0.99 (0.99-1.00)	0.4
Statin therapy	1.16 (0.88-2.75)	0.3
Active malignancy	1.84 (1.22-2.78)	0.003
High risk of fall	1.48 (1.12-1.96)	0.005

Table III. Multivariable analysis (model including CHA_2DS_2VASc score): predictive factors for intracerebral hemorrhage

	OR (95% CI)	P
Standard dose	Reference	
Non-label high dose NOACs	0.61 (0.21-1.80)	0.3
Label low dose	0.52 (0.37-0.72)	0.0001
Non-label low dose	0.48 (0.28-0.82)	0.008
CHA_2DS_2VASc score	1.02 (0.93-1.11)	0.6
Alcohol abuse	0.93 (0.62-1.38)	0.7
Hyperlipidemia	2.48 (1.85-3.34)	0.0001
Current smoker	0.89 (0.55-1.43)	0.6
Creatinine clearance	0.98 (0.97-0.99)	0.0001
Statin therapy	1.33 (0.96-1.81)	0.08
Platelet count	0.99 (0.99-1.00)	0.2
Concomitant antiplatelet therapy	3.37 (2.07-5.49)	0.0001
History of major bleeding	1.05 (0.68-1.61)	0.8
Active malignancy	1.46 (0.91-2.35)	0.1
High risk of fall	1.37 (1.00-1.87)	0.04

Table IV. Multivariable analysis (model including risk factors, white matter changes and excluding HAS-BLED and CHA_2DS_2VASc scores): predictive factors for intracerebral hemorrhage

	OR (95% CI)	P
Standard dose	Reference	
Non-label high dose NOACs	0.53 (0.17-1.62)	0.2
Label low dose	0.42 (0.30-0.60)	0.0001
Non-label low dose	0.47 (0.27-0.83)	0.009
Age	1.04 (1.02-1.06)	0.0001
Females	0.8 (0.60-1.05)	0.1
Hypertension	0.90 (0.60-1.35)	0.6
Diabetes mellitus	1.18 (0.86-1.61)	0.2
Hyperlipidemia	2.66 (1.96-3.61)	0.0001
History of myocardial infarction	0.88 (0.63-1.23)	0.4
Peripheral artery disease	1.23 (0.77-1.98)	0.3
Congestive heart failure	0.56 (0.40-0.79)	0.001
History of stroke/TIA	0.86 (0.65-1.14)	0.3
Alcohol abuse	1.00 (0.66-1.51)	0.9
Creatinine clearance	0.98 (0.97-0.99)	0.0001
Current smoker	1.14 (0.69-1.88)	0.6
History of major bleeding	1.04 (0.67-1.62)	0.8
Statin therapy	1.26 (0.90-1.75)	0.1
Platelet count	0.99 (0.99-1.00)	0.4
Concomitant antiplatelet therapy	3.54 (2.07-6.07)	0.0001
Active malignancy	1.46 (0.91-2.37)	0.1
High risk of fall	1.31 (0.95-1.80)	0.1
White matter changes*	2.28 (1.69-3.08)	0.0001

*for the forth model, was reported only the OR for white matter changes

Table V: First sensitivity analysis limited to cases and controls from the center of Perugia

Age	OR 1.02 (95% CI 1.01-1.04)
Hyperlipidemia	OR 2.58 (95% CI 1.74-3.14)
Concomitant use of antiplatelets	OR 3.97 (95% CI 2.24-7.02)
White matter changes	OR 2.33 (95% CI 1.74-3.14)
Clearance of creatinine	OR 0.98 (95% CI 0.97-0.99)
CHF	OR 0.58 (95% CI 0.40-0.83)

Table VI: Second sensitivity analysis limited to cases and controls from the center of Torino

Age	OR 1.11 (95% CI 1.08-3.15)
Hyperlipidemia	OR 1.74 (95% CI 1.17-2.60)
High risk of fall	OR 2.10 (95% CI 1.16-3.79)
White matter changes	OR 1.85 (95% CI 1.08-3.15)
Clearance of creatinine	OR 0.98 (95% CI 0.98-0.99)

Table VII. Characteristics of controls subdivided by centers

	Perugia (n=1022)	Torino (n=268)	Other Centers (n=236)
Age (years), mean*	79.2±8.4	65.7±9.7	73.6±10.1
Females*	500 (48.9%)	67 (30.0%)	110 (46.6%)
Apixaban	360 (35.2%)	84 (31.3%)	90 (38.1%)
Dabigatran	222 (21.7%)	63 (23.5%)	47 (19.9%)
Edoxaban	0	2 (0.8%)	13 (5.5%)
Rivaroxaban	440 (43.0%)	119 (43.4%)	85 (36.0%)
Creatinine clearance, mean	78.9±27.5	79.9±22.4	69.4±24.5
Hypertension*	922 (90.2%)	202 (75.4%)	196 (83.0%)
Diabetes Mellitus*	206 (20.1%)	41 (15.3%)	66 (27.9%)
Hyperlipidemia*	307 (30.0%)	102 (38.1%)	154 (65.2%)
Statin therapy*	279 (27.3%)	68 (25.4%)	151 (63.9%)
Alcohol abuse	171 (16.7%)	2 (0.8%)	14 (5.9%)
Congestive heart failure	252 (24.6%)	32 (12.0%)	64 (27.1%)
History stroke/TIA*	351 (34.3%)	32 (12.0%)	121 (51.3%
Myocardial infarction/angina pectoris	255 (24.9%)	37 (13.5%)	45 (19.0%)
Peripheral artery disease	58 (5.7%)	14 (5.2%)	25 (10.5%)
Paroxysmal AF*	296 (28.9%)	53 (20.0%)	211 (47.0%)
Concomitant antiplatelet therapy	26 (2.5%)	17 (6.3%)	8 (3.4%)
History of severe bleeding*	129 (12.6%)	4 (1.5%)	19 (8.0%)
Active malignancy	72 (7.0%)	1 (0.4%)	11 (4.7%)
Platelets per ml, mean	221000±76800	216000±53600	212000±60710

*p<0.05
